# Drug Synergy of Tenofovir and Nanoparticle-Based Antiretrovirals for HIV Prophylaxis

**DOI:** 10.1371/journal.pone.0061416

**Published:** 2013-04-22

**Authors:** Thanyanan Chaowanachan, Emily Krogstad, Cameron Ball, Kim A. Woodrow

**Affiliations:** Department of Bioengineering, University of Washington, Seattle, Washington, United States of America; Shanghai Medical College, Fudan University, China

## Abstract

**Background:**

The use of drug combinations has revolutionized the treatment of HIV but there is no equivalent combination product that exists for prevention, particularly for topical HIV prevention. Strategies to combine chemically incompatible agents may facilitate the discovery of unique drug-drug activities, particularly unexplored combination drug synergy. We fabricated two types of nanoparticles, each loaded with a single antiretroviral (ARV) that acts on a specific step of the viral replication cycle. Here we show unique combination drug activities mediated by our polymeric delivery systems when combined with free tenofovir (TFV).

**Methodology/Principal Findings:**

Biodegradable poly(lactide-*co*-glycolide) nanoparticles loaded with efavirenz (NP-EFV) or saquinavir (NP-SQV) were individually prepared by emulsion or nanoprecipitation techniques. Nanoparticles had reproducible size (d ∼200 nm) and zeta potential (-25 mV). The drug loading of the nanoparticles was approximately 7% (w/w). NP-EFV and NP-SQV were nontoxic to TZM-bl cells and ectocervical explants. Both NP-EFV and NP-SQV exhibited potent protection against HIV-1 BaL infection *in vitro*. The HIV inhibitory effect of nanoparticle formulated ARVs showed up to a 50-fold reduction in the 50% inhibitory concentration (IC_50_) compared to free drug. To quantify the activity arising from delivery of drug combinations, we calculated combination indices (CI) according to the median-effect principle. NP-EFV combined with free TFV demonstrated strong synergistic effects (CI_50_ = 0.07) at a 1∶50 ratio of IC_50_ values and additive effects (CI_50_ = 1.05) at a 1∶1 ratio of IC_50_ values. TFV combined with NP-SQV at a 1∶1 ratio of IC_50_ values also showed strong synergy (CI_50_ = 0.07).

**Conclusions:**

ARVs with different physicochemical properties can be encapsulated individually into nanoparticles to potently inhibit HIV. Our findings demonstrate for the first time that combining TFV with either NP-EFV or NP-SQV results in pronounced combination drug effects, and emphasize the potential of nanoparticles for the realization of unique drug-drug activities.

## Introduction

Sexual transmission is the primary cause of new HIV-1 infections worldwide, which today exceed 6,000 infections daily [1], [2]. Sub-Saharan Africa bears the burden of the global HIV/AIDS epidemic, and women in this region are disproportionately affected by the pandemic [3], [4]. Women account for three-quarters of Africans between the ages of 15 and 24 who are HIV-positive, and HIV/AIDS is the leading cause of death among women of reproductive age [3]. In the absence of an effective vaccine, and as long as new infections continue to outpace advances made in treatment with antiretroviral (ARV) drugs [5], biomedical prevention strategies are critical for stemming the spread of HIV. Oral pre-exposure prophylaxis (PrEP) and topical microbicides are the lead strategies for preventing HIV infection, but there is still a critical need for methods with greater efficacy to protect women [6]. Long-acting ARV drug combinations have the potential to enhance the efficacy of current ARV-based prevention strategies by overcoming low user adherence, and harnessing drug combinations with synergistic activity and breadth of coverage against the global diversity of HIV variants. However, the physicochemical diversity of ARV drugs precludes their co-formulation and limits access to all possible combinations of the >20 ARV drugs approved for clinical use [7], [8], [9]. Strategies that enable ARV drugs to be easily combined and provide sustained antiretroviral activity have the greatest potential to impact the efficacy of future biomedical prevention methods.

Nanocarrier systems provide an innovative approach for developing long-acting ARV drug combinations and have already been explored for use in HIV treatment and prevention [10], [11], [12], [13], [14]. The availability of different carrier systems, combined with the versatility of drugs that can be encapsulated for controlled release, motivate the use of nanocarrier systems for ARV drug delivery in different applications. In addition, nanocarriers have been shown in a number of examples to increase the activity and reduce cytotoxicity of several ARV drugs [15], [16], [17]. The selection of a nanocarrier system for drug delivery depends on the properties of the drug but also on the physical and chemical attributes of the final nanoformulation. The ability to control these attributes is important because the pharmacokinetics of the resulting nanoformulated drug can differ significantly from the parent compound. Properties of the delivery system such as carrier size, architecture and surface chemistry can also affect the activity of nanoformulated drugs. For example, Nowacek et al. demonstrated that physical characteristics of nanoparticles formed by wet-milling water-insoluble ARV drugs, i.e., particle size, surface charge, and shape, were correlated with macrophage uptake and resulted in greater antiretroviral efficacy [18]. The potential for rational design of drug carriers to enhance drug potency and efficacy may have important applications for prophylaxis.

Nanocarrier systems also have the capacity to address challenges associated with delivering drug combinations. The success of highly active antiretroviral therapy (HAART) provides a paradigm for developing the next generation ARV-based prevention strategies, giving rise to the possibility that a combination of potent and broadly active inhibitors will provide superior protection against HIV transmission and reduce the likelihood of emerging drug resistance. Drug combinations can also markedly expand the antiretroviral activity of single agents used alone by facilitating unique mechanisms of drug–drug activity when used in combination. For example, inhibition of drug metabolizing enzymes or efflux transporter systems has been implicated in the improved virological response to ARV drug combinations such as ritonavir in combination with saquinavir (SQV), and tenofovir (TFV) in combination with emtricitabine (FTC) or efavirenz (EFV) [19], [20], [21], [22]. However, due to physicochemical incompatibilities, not all ARV drugs are amenable to combinations that may lead to beneficial combination drug activity. As such, nanocarrier mediated ARV delivery may allow for the exploration of unique drug-drug interactions of otherwise incompatible ARV compounds or modulate drug delivery profiles necessary to achieve drug synergy using specific drug combinations.

Among ARV-based prevention methods, tenofovir (TFV) has been the most extensively investigated. TFV is a nucleotide analog reverse transcriptase inhibitor that is effective against either CCR5 or CXCR4 HIV-1, and has been shown to be safe for both oral and vaginal use [23], [24], [25], [26]. CAPRISA 004 was the first phase IIb double blind randomized controlled clinical trial to demonstrate protection against HIV-1 acquisition using TFV as a single ARV-based microbicide gel. In addition, oral TFV used either alone or in combination with emtricitabine (FTC) has been proven effective in three independent oral PrEP clinical trials (iPrEx, Partners PrEP, TDF2) [6]. Extensive safety and efficacy data exists for TFV, and it is furthest along in the development pipeline towards an ARV-based product that can protect against sexual HIV transmission. Improving the potency and long-acting efficacy of TFV by combining it with other ARV drugs is desirable for next generation biomedical prevention strategies. However, existing dosage forms may not be suitable for combining physicochemically diverse compounds and limits the realization of novel drug-drug interactions and synergies with TFV. Based on these observations, we sought to identify unique drug-drug activities mediated by our ARV loaded nanoparticles (NP-ARVs) when used in combination with free TFV.

Here we describe synergistic *in vitro* anti-HIV activity of novel combinations of NP-ARVs and free TFV. To overcome challenges associated with formulating multiple ARV compounds that are chemically incompatible, we fabricated polymeric nanoparticles encapsulating individual ARV drugs that were then delivered in combination. The non-nucleoside reverse transcriptase inhibitor (NNRTI) EFV and the protease inhibitor (PI) SQV were chosen based on their low aqueous solubility and different mechanisms of action. We show that EFV and SQV could be individually fabricated into biodegradable poly(lactide-*co*-glycolide) (PLGA) nanoparticles with high loading and encapsulation efficiency. NP-ARVs were nontoxic in cell culture and in mucosal tissue explants. In comparison to free ARVs, ARVs formulated in nanoparticles showed up to a 50-fold increase in antiviral activity. We also observed unique drug-drug activities when NP-ARVs were combined with free TFV, and observed in some cases drug synergy not seen with free drugs in combination. Collectively, our data show that PLGA-based nanoparticle formulations are a promising platform to deliver ARV combinations. The implications of our results may support a new paradigm for delivery of combination ARVs for HIV-1 prevention.

## Materials and Methods

### Materials

Poly(DL-lactide-*co*-glycolide) (PLGA) with molar ratios of 50∶50 was purchased from DURECT Corporation (Lactel - B6010-2P, MW ∼30 KD) and Sigma-Aldrich (Resomer - 502H, MW ∼30 KD). Chemical reagents for nanoparticle preparation were purchased from Fisher Scientific. Cell culture reagents (GIBCO, Invitrogen by Life Sciences Inc.) were used for the TZM-bl infectivity and cytotoxicity assays. The Promega™ Luciferase Assay System (Promega Co., Madison, WI) was used to determine luciferase protein expression. Tenofovir (TFV), efavirenz (EFV), and saquinavir (SQV) were obtained through the NIH AIDS Research and Reference Reagent Program (http://www.aidsreagent.org/).

### Fabrication of ARV loaded nanoparticles

Blank nanoparticles (vehicle control) and nanoparticles loaded with EFV or SQV were formulated individually. EFV loaded nanoparticles (NP-EFV) were formulated using a single emulsion technique as previously described [27], [28]. All concentrations described below are expressed in % w/v unless noted otherwise. In each preparation, EFV was dissolved in dichloromethane (DCM) containing 1.5% PLGA (w/v, Lactel - B6010-2P). Mass percentage of drug initially dissolved in PLGA (theoretical drug loading) was 15% (w/w). This mixture was then added drop-wise to an aqueous phase containing an emulsifier (5% aqueous solution of polyvinyl alcohol, PVA) to form an oil-in-water emulsion (o/w). A probe sonicator (3 mm diameter, Sonicator XL, Misonix, Farmingdale, NY) was used to homogenize the emulsion for 60 sec at 65 W. After solvent evaporation in an aqueous solution of 0.25% PVA for 3 h, nanoparticles were washed with deionized water three times by centrifugation at 14,000×g for 10 min (Sorvall Ultra 80, Waltham, MA). To formulate SQV loaded nanoparticles (NP-SQV), we used a nanoprecipitation technique [29]. SQV were dissolved in acetone containing 0.33% PLGA (w/v, Resomer - 502H) with 15% (w/w) theoretical drug loading. Then SQV-PLGA solution was added by syringe pump at a 1 mL/min flow rate to an aqueous solution containing 0.1% phosphate-buffered saline (0.01 M PBS, pH 7.4) and 0.1% dioctyl sulfosuccinate sodium (DSS) surfactant while it was stirring. Nanoparticles were formed instantly upon mixing due to the immiscibility of the polymer and non-solvent. After solvent evaporation, nanoparticles were washed as described above. The nanoparticles were suspended in deionized water and were lyophilized for 24 h under vacuum at 0.120 mbarr at −86°C (FreeZone 2.5 Plus, Labcono, Kansas City, MO). The dried nanoparticles were stored at −86°C until use.

### Characterization of nanoparticles

Size and zeta potential of the fabricated nanoparticles were determined using a Zetasizer Nano ZS90 (Malvern Instruments, AR). Size and morphology of nanoparticles were confirmed by scanning electron microscopy (SEM) visualized with a JEOL-7000 (JEOL Ltd, Sheboygan, WI) scanning electron microscope. Samples of nanoparticles were dusted onto carbon tape, coated with gold, and imaged using a 10 kV electron beam.

### Drug loading

Verification of drug-polymer association in nanoparticles was performed using Fourier transform infrared spectroscopy (FTIR). Briefly, 3–5 mg of nanoparticles were mixed with potassium bromide (KBr) using a mortar and pestle and analyzed in FTIR. Infrared spectra over a range of wavenumber 500 to 4000 cm^−1^ were monitored for the presence of the functional groups corresponding to the characteristic peaks of EFV or SQV. The amount of EFV and SQV actually loaded in nanoparticles was determined using a Shimadzu Prominence LC20AD high performance liquid chromatography (HPLC) system and LC Solutions software. A C18 column (Phenomenex, Torrance, CA), 5 µm, 250×4.6 mm i.d., was used for analysis with isocratic mode at a flow rate of 1.0 mL/min. Methods used to analyze EFV and SQV were based on those described previously for detecting SQV [30]. The mobile phase consisted of a mixture of 10 mM ammonium acetate buffer in HPLC grade water and acetonitrile at a 35∶65 ratio (v/v). SQV was detected at 238 nm and EFV was detected at 246 nm with retention times of 7.5 min and 8.6 min, respectively. Standard solutions of EFV and SQV were prepared at 1 mg/mL in dimethyl sulfoxide (DMSO) and diluted to generate the calibration curves. Analysis methods were validated with standard solutions and spiked samples. Linearity was established from 50 ng/mL to 10 µg/mL for EFV and 250 ng/mL to 50 µg/mL for SQV using a 10 µL injection volume. Nanoparticles were dissolved in DMSO to assess drug loading and encapsulation efficiency. For all *in vitro* assays, the delivered dose of the formulated ARV drug is defined and calculated based on the total drug loaded in the polymeric nanoparticle. Therefore, we delivered a mass concentration of the drug-loaded polymer to achieve the desired molar concentration of the drug given the volume requirements for the particular assay.

### *In vitro* release

To determine the release profiles of NP-ARVs in a physiological condition relevant to the vagina, an *in vitro* release study was conducted over 144 h using a vaginal fluid simulant (VFS) as the release medium [31]. Triplicate samples of approximately 2 mg of either NP-EFV or NP-SQV were suspended in 500 µL of VFS and added into individual dialysis tubes (1 kDa cut-off, GE Healthcare Bio-Sciences Corp., NJ). The dialysis tubes were placed in individual 50-mL tubes containing 5 or 15 mL of VFS for SQV and EFV, respectively, and incubated at 37°C on an orbital shaker at 100 rpm. At set time points (0.5, 1, 4, 8, 24, 48, 72, 144 h), 200 µL of samples were collected and replaced with fresh VFS. UV-HPLC methods were used to quantify the amount of drug in samples as described above.

### Cells, tissues and viruses

TZM-bl cells, PM1 cells, and HIV-1 BaL isolate were obtained through the NIH AIDS Research and Reference Reagent Program (http://www.aidsreagent.org/). TZM-bl cells are an engineered HeLa cells that express CD4, CCR5 and CXCR4 as previously described were used as reporter cells in the infectivity assay as described previously [32], [33], [34], [35]. Cells were maintained at 37°C, 5% CO_2_ in Dulbecco's Modified Eagle Medium (DMEM) with 10% fetal bovine serum, 1% 100X penicillin/streptomycin, and 1% 200 mM L-glutamine. PM1 cells were maintained at 37°C, 5% CO_2_ in RPMI 1640 with 10% fetal bovine serum, 1% 100X penicillin/streptomycin, and 1% 200 mM L-glutamine and were used for preparing HIV-1 viral stock [36].

### Cellular viability assay

TZM-bl cells were seeded in a 96-well plate at 5,000 cells/well and incubated overnight to allow the cells to adhere to the well. Dilutions of drugs (free and nanoparticle forms) were added to triplicate wells of TZM-bl cells. Wells containing cells alone served as controls. Cell cultures in the absence and presence of drugs were incubated for 48 h. To determine cell culture viability, metabolic capacity of cells was measured using the Promega CellTiter-Blue™ assay according to the manufacturer's instructions. The ability of cells to reduce a resazurin indicator dye to fluorescent resorufin was measured using a plate reader at 560/590 nm (excitation/emission) and normalized to media only-treated cells (100% viability). DMSO served as positive control.

### Antiviral activity

The inhibitory activity of free and nanoparticle ARVs against HIV-1 BaL was determined in TZM-bl cells by luciferase quantification of cell lysates [37], [38]. Cells were seeded at 5,000 cells/well and grown to approximately 50–60% confluence overnight prior to infection. Dilutions of each drug were added to triplicate wells. After 1 h, HIV-1 BaL at an approximate TCID_50_ (50% tissue culture infectious dose) of 300 per well was added to each pre-treated well. Media controls (wells containing cells alone) were included in every run for luminescent background subtraction. Cells grown in the absence of virus served as the negative infectivity control (100% inhibition), while cells infected with virus in the absence of drug served as the positive infectivity control (0% inhibition). After 48 h, cells were lysed and luciferase expression was determined in relative light units (RLUs) using a luminometer. The percent inhibition was calculated for all test and control cultures to determine the 50% inhibition concentration (IC_50_) value of each drug. The IC_50_ values of NP-ARVs were calculated using drug concentrations that corresponded to the actual drug loading determined by HPLC.

### Combination effect

**Figure 1:**
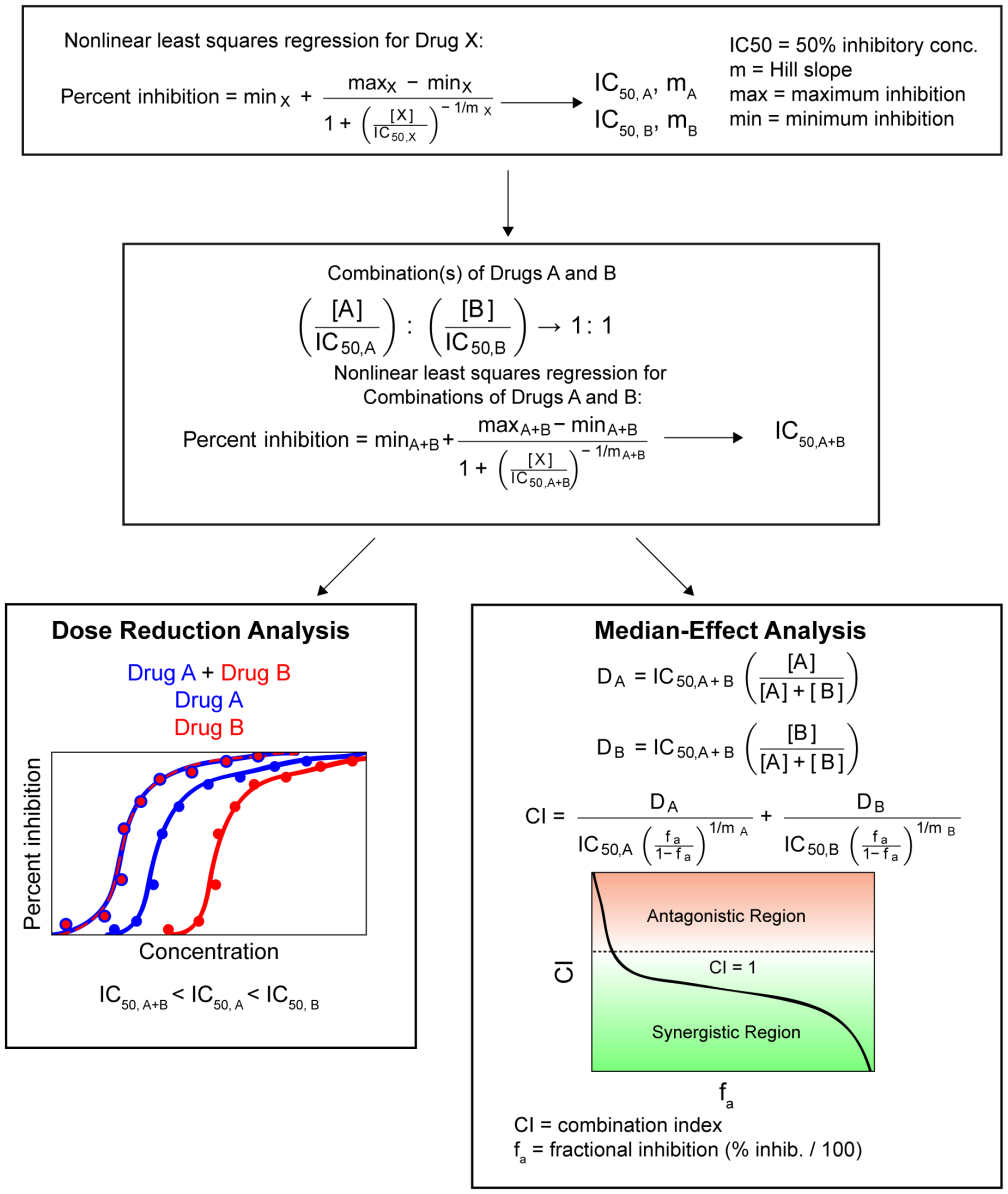
Schematic diagram for combination effect analysis. Drug combinations were analyzed for their ability to affect dose reduction and synergy. First, individual drugs, either free or encapsulated, were used to create dose response curves using the TZM-bl assay. These curves were fit to a sigmoid curve using nonlinear least squares regression to estimate drug IC_50_ and the Hill slope. Next, combinations of drugs at their equipotency ratio (1∶1 ratios of IC_50_ values) were used to create similar curves using the TZM-bl assay. These were also fit to a sigmoid curve using nonlinear least squares regression to estimate the IC_50_ and Hill slope of the combination. Finally, comparison of IC_50_ values combinations were used to estimate dose reduction. The median-effect analysis was performed to measure combination effects.

Combined activity of dual drug combination was evaluated as described in Figure 1. First, median IC_50_ values of each drug were obtained using the TZM-bl antiviral activity assay as described above. Next, each drug was added to free TFV at a 1∶1 ratio based on their IC_50_ values to create a mixture of combination drugs. For NP-ARVs, amounts of the individual agents used in combinations were determined using the measured drug loading. The drug mixtures were serially diluted and IC_50_ values were determined with the TZM-bl assay. Finally, combination effects were evaluated by; 1) comparing IC_50_ values to determine dose reduction; and 2) identifying combination indices (CI) to quantify drug synergy, with the median-effect analysis described by Chou and Talalay [39]. The CI of each drug combination was plotted as a function of the fractional inhibition (Fa) by computer simulation from Fa = 0.10 to 0.95. In this analysis, the combined effect at the 50% fractional inhibition (CI_50_) was reported as synergistic, additive, or antagonistic when CI<1, = 1, or >1, respectively.

### *Ex vivo* toxicity assay

Ectocervical tissues from two macaques (Tissue Banking and Distribution Program, the Washington National Primate Research Center) were processed for polarized explant cultures as previously described [40], [41], [42]. The explant cultures were set up on the day of surgery. Briefly, the macaque ectocervical explant cultures were established in duplicate by inserting a circular tissue punch through a hole in a transwell with the luminal epithelium side up. The edges around the explant were sealed with Matrigel™ (BD Biosciences, San Jose, CA). A 0.1 mg/mL suspension of either NP-EFV or NP-SQV in 200 µL of culture media (DMEM with 10% fetal bovine serum, 1% 100X penicillin/streptomycin, and 1% 200 mM L-glutamine) was added on the apical side of the tissue. Untreated explants (culture media) and explants treated with 0.4% nonoxynol-9 (N-9) gel served as controls. All explant cultures were maintained at 37**°**C in a 5% CO_2_ atmosphere. After 18–24 h, the explants were washed and one of each duplicate was incubated in RPMI containing 250 µg/mL MTT [1-(4,5-dimethylthiazol- 2-yl)-3,5-diphenylformazan] for 4 h. The explants were removed and placed in 1 mL of methanol overnight to extract the formazan dye produced by live tissue. The next day, the explants were removed from methanol and placed on a paper towel to dry and be weighed. The color extracted in methanol was read for optical density at 595 nm. Percent viability of the treated explants was determined by correcting the optical density (OD) with the weight of the corresponding explant. The other explant was frozen in an embedding medium (Tissue-Tek, Sakura Finetek USA Inc., CA) and processed for histology by cryosectioning and hematoxylin-eosin staining by Comparative Pathology Program/Histology and Imaging Core Research Laboratory, University of Washington School of Medicine at South Lake Union, Seattle, WA.

### Statistical and mathematical analyses

The IC_50_ values were calculated using a four-parameter sigmoid regression and bootstrapping (MATLAB R2010b software, MathWorks, Natick, Massachusetts) as previously described [43]. Briefly, confidence intervals were determined using a sampling procedure that created data sets by random sampling with replacement for curve fits 1000 times. The IC_50_ values for each drug alone and in combination are presented as median IC_50_, 95% confidence interval (C.I.) based on percentiles from a histogram of IC_50_ values, and the coefficient of variation (Cv). The combination effect was analyzed using MATLAB_R2010b software.

## Results

### ARVs are effectively formulated into polymeric nanoparticles

We demonstrate that ARV compounds with low aqueous solubility can be formulated into PLGA nanoparticles with reproducible size, shape, and high drug loading content. We chose EFV and SQV as model drugs with low aqueous solubility (<0.1 mg/mL) that may be challenging to combine with TFV, especially for topical microbicide applications. The calculated value of the partition coefficient (logP) and aqueous solubility are useful parameters to determine the physicochemical properties of the ARVs [44]. The logP values of EFV and SQV are 3.8–4.5, and their aqueous solubility at 25°C are 8.55 µg/mL and 2.47 µg/mL, respectively [45]. Despite the similar logP and aqueous solubility, EFV and SQV required different methods for encapsulation into PLGA nanoparticles. For NP-EFV, a single emulsion-solvent evaporation technique was employed wherein the EFV and polymer were combined in DCM and aqueous PVA was used as a surfactant. NP-SQV formulated by the same technique resulted in low loading (<1% w/w) (data not shown). Therefore, NP-SQV were fabricated by a nanoprecipitation method in which two miscible solvents are employed to induce the precipitation of the drug and polymer mixture. Nanoprecipitation allows for instantaneous particle formation due to the miscibility of the polymer solvent and non-solvent, compared to the slower particle hardening process that occurs with the single emulsion process [27], [29]. We also modified the formulation process by using acetone and adjusting the solvent/non-solvent ratio to prevent partition of SQV to the aqueous phase.

**Figure 2:**
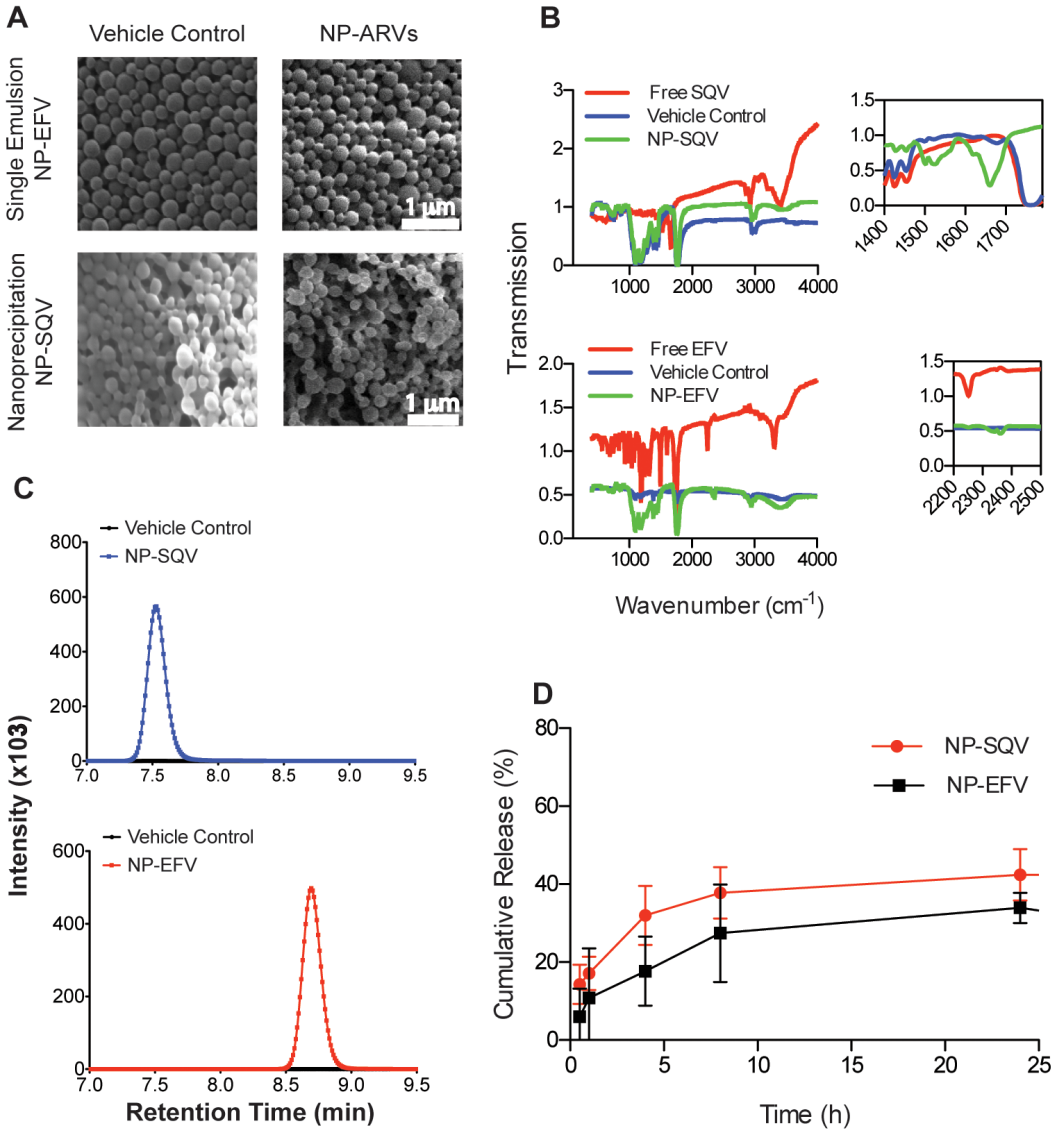
Properties of PLGA nanoparticles loaded with efavirenz or saquinavir. (A) Scanning electron photomicrographs (magnification, 15,000×) of nanoparticles encapsulated with antiretroviral drugs efavirenz (NP-EFV) or saquinavir (NP-SQV). (B) Fourier transform infrared spectroscopy (FTIR) confirmation of the antiretroviral drugs loaded into PLGA nanoparticles. Insets show characteristic frequencies of SQV and EFV and the PLGA polymer (Vehicle Control). (C) HPLC chromatograms of vehicle control (black), SQV (blue) and EFV (red) nanoparticles showing the detection of SQV and EFV only in ARV loaded nanoparticles. No drug peak was detected in the vehicle control nanoparticles. (D) Cumulative release of EFV and SQV from nanoparticles in a vaginal fluid simulant (VFS) showing the release of SQV and EFV over 24 h.

Table 1 lists properties of NP-EFV and NP-SQV fabricated with emulsion and nanoprecipitation techniques, respectively. Both NP-EFV and NP-SQV showed a large negative zeta-potential of approximately −25 mV, a value predictive of high colloidal stability due to large repulsive charges [46]. NP-EFV had a particle size of approximately 200 nm with low polydispersity (<0.08). However, in some cases, NP-SQV showed two distinctly sized populations, indicating bimodal distribution. One population had a mean diameter of ∼100–200 nm, and we detected a second population with a mean diameter of ∼600–2500 nm. We used scanning electron microscopy (SEM) to confirm the size and morphology of nanoparticles. SEM micrographs revealed that both NP-EFV and NP-SQV were spherical with an average particle diameter of ∼200 nm (Figure 2A). Owing to the SEM results, we expect the bimodal distribution observed with DLS can be attributed to a population of nanoparticle aggregates in aqueous suspension. Our findings are similar to those describing manufacturing of PLGA nanoparticles via emulsion and nanoprecipitation techniques [28], [29], [37], [47], [48]. Our results suggest that these two techniques are suitable for encapsulating hydrophobic drugs.

**Table 1:** Physicochemical properties of nanoparticles loaded with anti-HIV agents.

Drug^a^	Theoretical Drug Loading (% w/w)	Size^b^ (d.nm. ± SD)	PDI^b^	Zeta Potential^b^ (mV ± SD)	Actual Drug Loading (% w/w, ± SD)	Encapsulation Efficiency (%, ± SD)
**EFV**	15	227±1.8	0.05	−24.4+7.3	6.7±0.4	44.5±2.7
**SQV**	15	189±96.3^c^	0.486	−24.2±1.5	7.2±2.2	48.3±15.2

a Efavirenz (EFV) and Saquinavir (SQV) nanoparticles were synthesized using single emulsion and nanoprecipitation techniques, respectively.

b The particle size, polydispersity indices (PDI) and zeta potential were determined using dynamic light scattering (DLS); data are the average of three batches.

c Particles showed two peak sizes. This number represents the average peak intensity size for three batches of SQV nanoparticles. Two of the three batches displayed bimodal size distribution with one peak ∼100–200 nm and another peak at ∼600–2500 nm. Since the large peaks are likely indicative of aggregated clumps of particles and not individual particles, these large peaks were not included in the average size shown here but still affect the PDI value.

FTIR spectroscopy and UV-HPLC was used to confirm drug loading into polymer nanoparticles. FTIR absorption spectra for NP-ARVs detected characteristic bond vibrational frequencies for both the drug compound and the PLGA polymer. Infrared absorbance spectra of NP-SQV demonstrated characteristic frequencies of the phenyl (1500 cm^−1^) and amide carbonyl (1695 cm^−1^) present in the drug, as well as the ester stretching frequency (1750 cm^−1^) indicative of the polymer (Figure 2B). FTIR spectra of NP-EFV showed absorption bands at 2300 cm^−1^ from the alkyne and at 600–800 cm^−1^ from the C-Cl alkyl halide stretching, in addition to the ester band from the PLGA polymer. These FTIR results strongly indicate drug loading within the polymer nanoparticles.

To quantify actual drug loading and encapsulation efficiency, we employed established methods for detection and separation of EFV and SQV from excipients of the nanoparticle formulation process using UV-HPLC. We demonstrate that nanoparticles prepared with a theoretical drug loading of 15% (weight of ARV to weight of polymer, w/w) achieved average actual drug loading of approximately 7% (w/w) and encapsulation efficiency of approximately 50% (Table 1). We validated our method for dissolution of the polymer matrix to release the drug for detection using vehicle control nanoparticles (no drug) spiked with known quantities of drug. These validation experiments indicated a high recovery (97–99%) and demonstrated the accuracy of our methods to quantify drug loading. As shown in Figure 2C, SQV and EFV were detected only in ARV loaded nanoparticles, whereas no compounds of similar retention time were detected in the vehicle control nanoparticles. NP-EFV had a comparatively high drug loading of 6.7±0.4% (w/w) sufficient for use in our *in vitro* efficacy studies [49], [50], [51]. Using the nanoprecipitation technique, we obtained NP-SQV that had 24 times higher drug loading and encapsulation efficiency of ∼50% (Table 1) compared to NP-SQV formulated using a single emulsion technique.

To determine if we could achieve higher encapsulation efficiencies, we also prepared nanoparticles with a lower initial drug loading of 5–7.5% (w/w). For NP-EFV, we observed that decreasing the theoretical drug loading decreased the actual loading of EFV, but had no effect on the encapsulation efficiency. NP-EFV with 15% (w/w) theoretical drug loading improved the actual drug loading by 2-fold compared to preparing particles with 5% (w/w) theoretical drug loading (actual drug loading = 3.0±0.45% w/w). In contrast to NP-EFV, we observed that reducing the initial amount of SQV used in the nanoprecipitation process doubled the encapsulation efficiency without reducing the drug loading. The actual drug loading of NP-SQV was independent of the initial loading in the range tested. NP-SQV with a theoretical drug loading of 7.5% (w/w) or 15% (w/w) had similar actual drug loading of 6–7% (w/w).

### Nanoparticles modulate ARV release

We investigated the drug release profile of NP-ARVs using a vaginal fluid simulant (VFS) that mimics the composition and viscoelastic properties of cervico-vaginal secretions produced by healthy, non-pregnant, premenopausal women [31]. UV-HPLC was used to measure drug release from nanoparticles into VFS over 144 h. The *in vitro* release of both EFV and SQV from nanoparticles followed a biphasic release profile, where an initial burst release of 10–20% of drug was observed within 1 h followed by sustained drug release over 24 h. During the first 24 h, we observed a total cumulative release of 33.9±3.9% and 42.4±6.6% for EFV and SQV, respectively (Figure 2D). Although we measured drug release out to 144 h, we did not detect a significant accumulation of drug release after 24 h. The percent cumulative release at 24 h corresponds to a mass ratio of 0.022 mg EFV/mg PLGA and 0.025 mg SQV/mg PLGA released at 24 h. Based on drug loading and release results, as well as the reported IC_50_ values for free ARVs, we estimated that delivering approximately 10^−3^ mg/mL of NP-SQV or 10^−6^ mg/mL of NP-EFV would be sufficient to observe *in vitro* protection using the TZM-bl assay.

### NP-ARVs are nontoxic to *in vitro* cell line and *ex vivo* ectocervical explants

**Figure 3:**
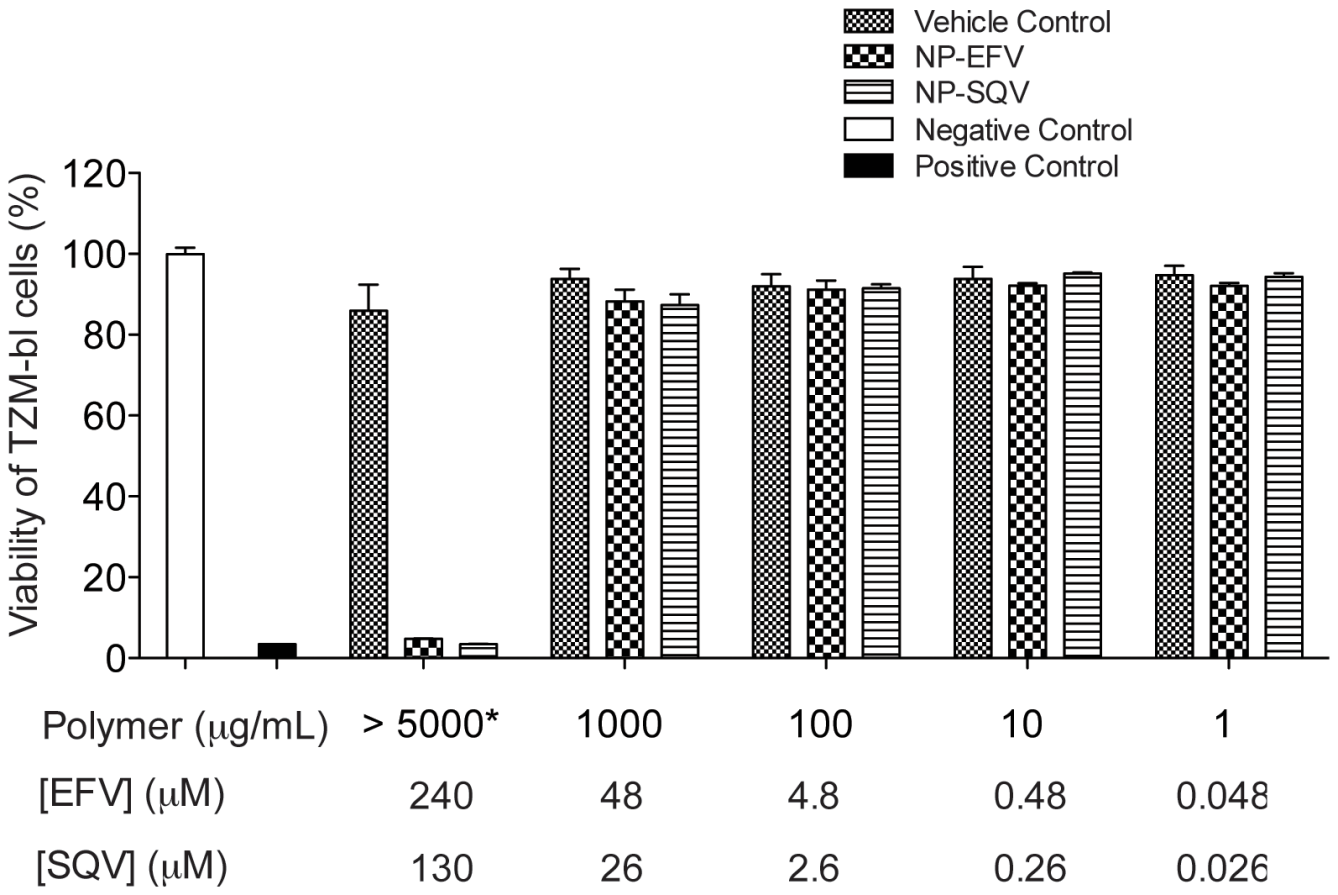
PLGA nanoparticles loaded with EFV or SQV show low cytotoxity. Viability of TZM-bl cells measured by the CellTiter-Blue™ (Promega) viability assay demonstrating non-toxic concentrations (>80% viability) of efavirenz nanoparticles (NP-EFV) and saquinavir nanoparticles (NP-SQV) at ≤1,000 µg of polymer/mL (≤48 µM EFV and ≤26 µM SQV). Vehicle control nanoparticles at the concentrations tested showed no reduction of viability (100%±8%), indicating non-cytotoxicity of PLGA polymer. Negative control = media only, Positive control = DMSO. *Vehicle control for NP-SQV was measured at 10,000 µg of polymer/mL.

PLGA nanoparticles loaded with EFV or SQV were neither cytotoxic to cells nor tissue explants over the range of concentrations evaluated. We evaluated cytotoxicity of our NP-ARVs in TZM-bl cell culture before testing their bioactivities to exclude effects of nanoparticles on the viability of TZM-bl cells. Cytotoxicity of our NP-ARVs was measured over a range of polymer concentrations from 1 to 10,000 µg/mL after 48 h of exposure. Compared with the negative control (media only), vehicle control nanoparticles at concentrations ≤5 mg polymer/mL showed no reduction of viability (100%±8%), suggesting that PLGA nanoparticles alone are not cytotoxic below these concentrations. We observed >80% viability of TZM-bl cells for NP-EFV and NP-SQV tested at ≤1,000 µg of polymer/mL (≤48 µM EFV and ≤26 µM SQV) (Figure 3). Both NP-EFV and NP-SQV showed cytotoxicity at concentrations >5 mg polymer/mL (>240 µM EFV and >130 µM SQV). Since anti-HIV bioactivity was measured at doses well below polymer concentrations that were cytotoxic, we did not expect toxicity to confound the outcome of the antiviral activity assays.

**Figure 4:**
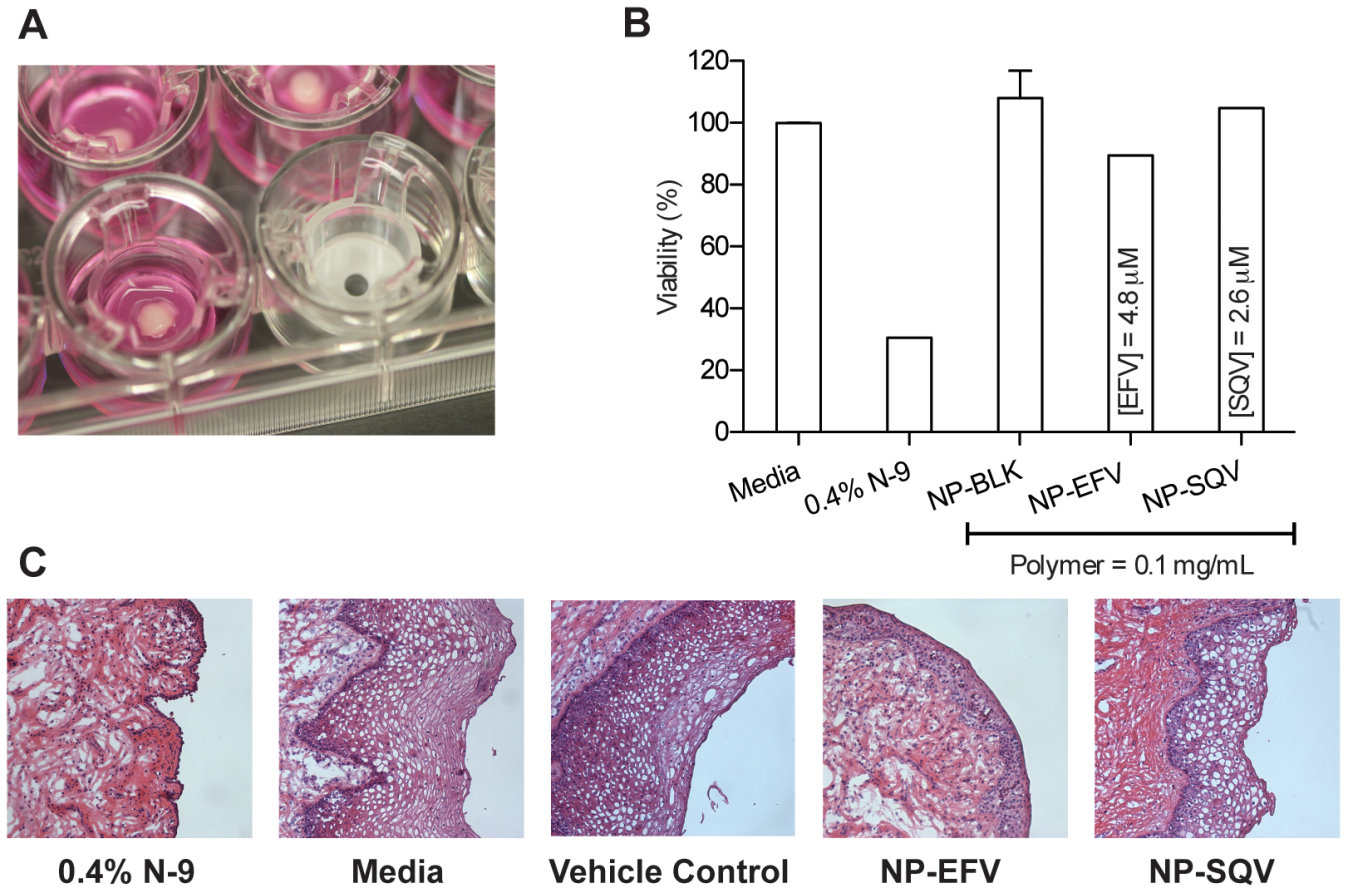
Ectocervical explants confirm the safety of NP-ARVs. Viability of explants from two macaques was assessed at 18–24 h after application using an MTT assay and histology. (A) A circular tissue punch of macaque ectocervical explants was inserted through a transwell membrane and placed to assure exposure to products (luminal epithelium up). (B) Viability of explants measured by the MTT assay demonstrating viability of explant tissue exposed to nanoparticles loaded with efavirenz (NP-EFV, n = 1) or saquinavir (NP-SQV, n = 1). Tissue viability was similar to media control (untreated explants, n = 3) while the toxicity control (nonoxynol-9 (N-9) treated explant, n = 1) showed significant reduction in tissue viability. (C) Histological photographs of macaque ectocervical explants (hematoxylin and eosin stain; magnification, ×100) show no visual changes following exposure to both NP-EFV and NP-SQV treated explants, and the destruction of the epithelial layer of an N-9-treated explant.

To confirm the results obtained with *in vitro* cytotoxicity in TZM-bl cells, we evaluated the safety of our NP-ARVs using polarized explant cultures. We chose to test nanoparticles (NP-ARVs or vehicle control) at a 0.1 mg/mL dose. This dose was shown to be nontoxic to TZM-bl cells and is several-fold higher than doses required for efficacy *in vitro*. We used two explant tissues per treatment – one for the MTT assay and the other for histology. For controls, explants were treated with either 0.4% nonoxynol-9 (N-9) or media (untreated). We evaluated explants for viability and tissue morphology at 18–24 h after application. The N-9-treated explants showed significant reduction in tissue viability, as measured by the MTT assay (n = 1), and destruction of the epithelial layer was observed by histology (n = 1) (Figure 4). The toxicity of N-9 found in our study was consistent with previous studies performed using human explant tissues [40], [41]. Viability of explants determined by the MTT assay showed that neither NP-EFV (n = 1) nor NP-SQV (n = 1) altered tissue viability compared to the media control (untreated explants, n = 3) (Figure 4B). Histological analysis of the ectocervical explants after 24 h of exposure to NP-ARV produced no visual changes to the integrity of epithelial layer (Figure 4C). The results with ectocervical tissue explants confirmed the findings obtained using the TZM-bl cytotoxicity model, suggesting that our NP-ARV are nontoxic to the cells and are a biocompatible vehicle for drug delivery to the mucosal tissue, particularly the ectocervical tissue of the lower female reproductive tract.

### NP-ARVs potently inhibit HIV-1 BaL infection

To ensure ARVs loaded into nanoparticles retained reproducible bioactivity against HIV-1, we tested three batches of NP-EFV using the TZM-bl assay. As described in the methods, a mass concentration of the drug-loaded polymer was delivered to achieve the desired molar drug concentration in the assay volume irrespective of the observed drug release kinetics. We observed consistent activity between three independent batches of NP-EFV to inhibit HIV-1 BaL at nanomolar levels, with an average IC_50_ value of 0.52±0.17 nM (mean ± SD, n = 3). This value is lower than previously reported IC_50_ values of unformulated (free) EFV, indicating no loss in drug activity due to the formulation processes [52].

**Figure 5:**
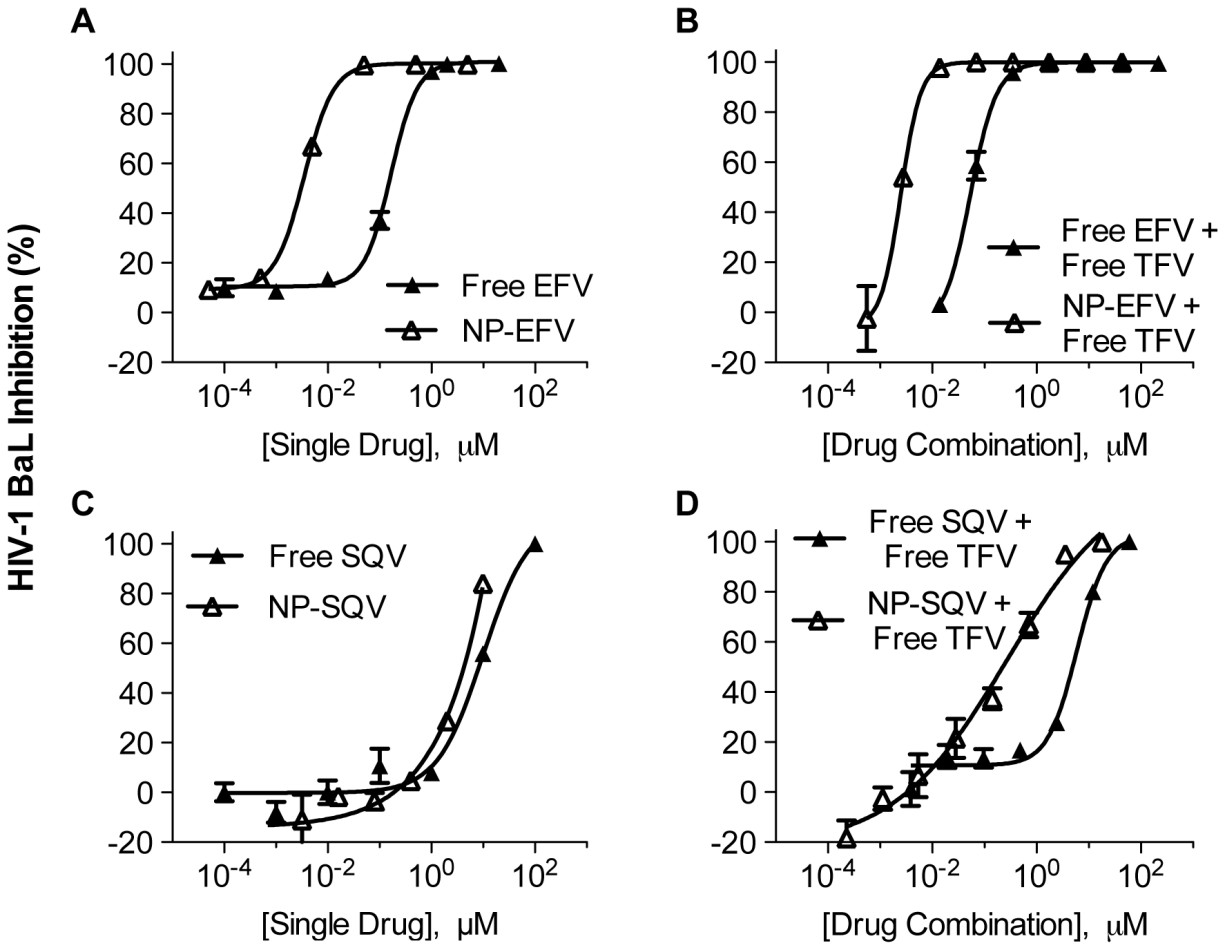
NP-ARVs alone and in combination with free TFV show potent antiviral activity. Tenofovir (TFV) alone and in combination with efavirenz (EFV) or saquinavir (SQV) was investigated in TZM-bl cells. Data represent mean values obtained from triplicate infections. The dose-response curves show antiviral activity of free EFV, free SQV, EFV and SQV loaded nanoparticles (NP-EFV and NP-SQV) alone (A and C) or in combination with free TFV (B and D). At a 1∶11 molar ratio (1∶1 ratio of IC_50_ values of free EFV and free TFV, the antiviral activity of free TFV combined with NP-EFV showed a 3-fold reduction in the IC_50_ value compared to the free drug combinations (B). The antiviral activity of free TFV combined with free SQV or NP-SQV was tested at a 1∶1 ratio of IC_50_ values (1∶5 TFV∶SQV and 1∶3 TFV∶NP-SQV molar ratio). Free TFV combined with NP-SQV showed a 20-fold reduction in the IC_50_ value compared to the free drug combinations (D).

We further evaluated antiviral activities of NP-ARVs in comparison with their free forms. After exposure of TZM-bl cells to NP-ARVs or free ARVs, we observed potent antiviral activity against HIV-1 BaL with estimated IC_50_ values in the nanomolar and micromolar ranges for EFV and SQV, respectively (Table 2). Compared with free EFV, NP-EFV showed higher HIV inhibitory activity, with a 50-fold reduction in IC_50_ (Table 2 and Figure 5A). NP-SQV also showed higher HIV inhibitory activity when compared with free SQV, with a nearly 2-fold reduction of the IC_50_ (Table 2 and Figure 5C). We observed that blank nanoparticles (vehicle control) tested at the same ranges of polymer concentrations showed no HIV inhibition and were comparable to the negative media control (<5%). This indicates that PLGA nanoparticles alone do not provide inhibition against HIV-1 infection. Together, our results suggest that ARVs loaded into nanoparticles possess potent bioactivity that is superior to that of unformulated ARVs. Since PLGA nanoparticles are known to enhance internalization and intracellular uptake [49], [53], [54], [55], we hypothesize that the enhanced potency of our NP-ARVs formulated with PLGA may be due to higher intracellular ARV drug concentration.

**Table 2:** Summary of 50% inhibitory concentrations (IC_50_) determined by infecting TZM-bl cells with HIV-1 BaL.

Free ARV Alone or Combined with Free Tenofovir				Nanoparticle-ARV (NP-) Alone or Combined with Free Tenofovir			
Drug(s)	IC_50_ (median)	95% C.I.^a^	C_V_ a	Drug(s)	IC_50_ (median)	95% C.I.^a^	C_V_ a
**Free TFV**	1.81 µM	[1.45, 2.33] µM	0.14	**Free TFV**	1.81 µM	[1.45, 2.33] µM	0.14
**Free EFV**	0.164 µM	[1, 204] nM	0.320	**NP-EFV**	0.003 µM	[1.97, 4.81] nM	0.301
**Free SQV**	9.79 µM	[9.25, 11.3] µM	0.047	**NP-SQV**	6.08 µM	[5.84, 6.21] µM	0.016
**Free EFV + Free TFV** b	0.01 µM	[0.01, 0.014] µM	0.189	**NP-EFV + Free TFV** c	0.003 µM	[0.002, 0.003] µM	0.114
**Free SQV + Free TFV** b	5.95 µM	[0.192, 7.22] µM	0.195	**NP-SQV + Free TFV** b	0.27 µM	[0.17, 0.39] µM	0.226

a Data show the confidence interval (C.I.) based on the 2.5 and 97.5 percentiles of bootstrapped IC_50_ estimates, and the coefficient of variation (Cv) of the IC_50_ of each drug alone and in combination.

b Combined activities were evaluated using a 1∶1 ratio of IC_50_ values, corresponded to the molar ratios of 1∶11 Free EFV∶Free TFV, 1∶5 Free TFV∶Free SQV, and 1∶3 Free TFV∶NP-SQV.

c Combined activities were evaluated using a 1∶50 ratio of IC_50_ values, corresponded to the molar ratios of 1∶11 Free EFV∶Free TFV.

### NP-ARVs showed potent HIV inhibitory activity alone and in combination with TFV

We conducted drug combination studies to identify unique drug-drug activities facilitated by our nanoparticle delivery systems. The activity of drug combinations is typically assessed at their equipotency ratio (1∶1 ratios of IC_50_ values), which is the ratio at which the contribution of each drug to the combined efficacy is estimated to be equal [39]. However, there is no established precedence for conducting combination studies with nanoparticle-formulated drugs. Therefore, in addition to the equipotency ratio, we tested other ratios of the NP-ARVs in combination with free TFV.

The dose-response relationships showing the inhibitory activities of free TFV alone and in combination with either EFV or SQV (both free and nanoparticle formulations) against HIV-1 BaL are presented in Figure 5 and Table 2. Free drug combinations of EFV and TFV at their equipotency ratio (1∶1 ratio of IC_50_, or 1∶11 EFV∶TFV molar ratio) had greater anti-HIV activity and showed a lower value of the IC_50_ measured for either of the individual ARVs used alone. For example, the combination of free TFV and free EFV produced an IC_50_ value of 10 nM, whereas the IC_50_ values for free TFV and free EFV used alone were 1.8 µM and 0.2 µM, respectively. Therefore, the combination of the free drugs was 20–200 times more potent than either of the single ARVs. However, NP-EFV combined with free TFV at their equipotency ratio (1∶1 ratio of IC_50_, or 1∶600 NP-EFV∶TFV molar ratio) showed an IC_50_ (∼1.0 µM) that was intermediate in value between free TFV (1.8 µM) and NP-EFV (∼3.0 nM) used alone. As such, in contrast to the enhanced potency obtained from combining the free drugs, and despite the 50-fold enhanced potency of NP-EFV compared to free EFV, the combination activity of NP-EFV and free TFV appeared to be dominated by the activity of free TFV alone. This result may be unsurprising because, at a molar ratio of 1∶600 of NP-EFV∶TFV near the IC_50_ value, the number of nanoparticles per cell is <10 whereas the number of molecules of TFV per cell is >10^14^. In the limit where the nanoparticle number is small relative to the number of cells, significant heterogeneity within the system may confound the true measure of combination activity of multiple drugs. Based on this observation, we combined NP-EFV and free TFV at the same molar ratio used to achieve equipotency of the free drugs (1∶11 EFV∶TFV molar ratio). At this ratio, we found that NP-EFV in combination with free TFV had a 3-fold reduction in the IC_50_ value compared to the free drug combinations (Figure 5B).

The combination activity of NP-SQV with free TFV also demonstrated favorable dose reduction that was superior to the combined activity of free SQV with free TFV (Figure 5D). The antiviral activities of free TFV combined with either free SQV or NP-SQV were tested at their equipotency ratio, which corresponded to molar ratios of 1∶5 TFV∶SQV and 1∶3 TFV∶NP-SQV. We found that the combination of free TFV and free SQV did not significantly enhance antiviral potency as was observed when free TFV was combined with free EFV. The IC_50_ measured for the combination of free TFV and free SQV was intermediate in value compared to the IC_50_ measured for the individual ARVs (Table 2). Compared to free TFV alone, the IC_50_ value for the combination of free TFV and free SQV increased by approximately 3-fold (5.95 µM vs. 1.81 µM). This indicates that the inhibitory activity of free TFV combined with free SQV was lower than that of free TFV alone. However, when compared to free SQV alone, the IC_50_ value for the combination of free drugs decreased approximately 2-fold (9.79 µM vs. 5.95 µM). This result suggested that the inhibitory activity of combined drug was greater than that of free SQV alone. In contrast to the combination free TFV and free SQV, free TFV combined with NP-SQV showed greater inhibitory activity than that of free TFV and free SQV alone. We found that combination of free TFV and NP-SQV resulted in a 20-fold reduction in the IC_50_ value (∼3 nM) compared to the free drug combinations (∼6 µM). Collectively, the dose reduction results indicated that NP-EFV and NP-SQV used alone or in combination with free TFV showed greater potency than the free drug equivalent.

### NP-ARVs combined with free TFV exhibit strong synergistic activity

**Figure 6:**
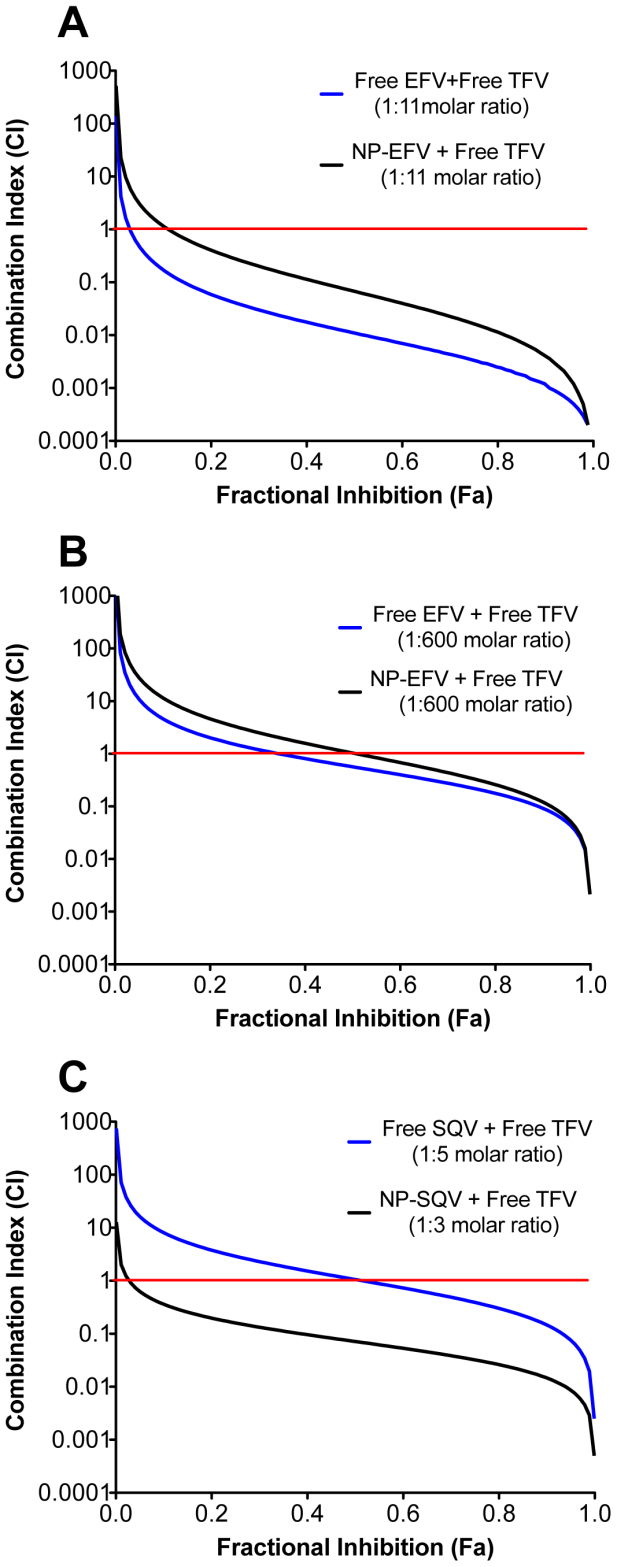
TFV combined with NP-EFV or NP-SQV showed strong synergism. Combination effects of free tenofovir (TFV) with efavirenz (EFV) or saquinavir (SQV) were quantified using the TZM-bl infectivity assay and the HIV-1 BaL isolate. The combination index (CI) was determined as described by Chou and Talalay. CI<1, = 1, and >1 indicate synergistic, additive, and antagonistic effects, respectively. The red line at CI = 1 represents the additive effect. (A) Combination of free TFV with free EFV or with nanoparticles loaded with EFV (NP-EFV) at a 1∶11 EFV∶TFV molar ratio demonstrated strong synergism, with the CI at 50% inhibition (CI_50_) of 0.01 and 0.07, respectively. (B) Combination of free TFV with free EFV or with NP-EFV at a 1∶600 NP-EFV∶TFV molar ratio demonstrated synergism and addition, with the CI_50_ of 0.58 and 1.05, respectively. (C) Combination of free TFV with free SQV at a 1∶5 TFV∶SQV molar ratio showed an additive effect (CI_50_ = 1.04) while free TFV combined with nanoparticles loaded with SQV (NP-SQV) at a 1∶3 TFV∶NP-SQV molar ratio showed a synergistic effect (CI_50_ = 0.07).

The CI was determined for ARV drugs combined at molar ratios to achieve equipotency (1∶1 ratios of IC_50_ values). The CI of each free drug or NP-drug combination was plotted as a function of the fractional inhibition (Fa) from 0.10 to 0.95 (Figure 6). The plots show symmetric curves of fractional inhibition and combination indices (log CI), demonstrating that synergism and antagonism can vary depending on fractional inhibition values. For example, the CI plot of free SQV combined with free TFV demonstrated an additive effect at the 50% fractional inhibition (CI_50_ = 1.04) and synergistic effects with CI_70_ and CI_95_ values of 0.8 and 0.2, respectively (Figure 6C). We interpreted the combination effects at the CI_50_ value, and defined drug synergy as a CI_50_ value less than 1.

Free EFV and NP-EFV combined with free TFV at their respective equipotency ratio (1∶1 ratio of IC_50_ values) demonstrated a synergistic (CI_50_ = 0.01) and additive (CI_50_ = 1.05) effect, respectively (Figure 6A and 6B). However, when combined at the equipotency ratio used for the free drugs (1∶11 molar of EFV∶TFV), NP-EFV and TFV demonstrated a strong synergistic effect with a measured CI_50_ value of 0.07 (Figure 6A). The free drug combinations of EFV and TFV were also measured at the equipotency ratio used for NP-EFV and free TFV (1∶600 molar ratio, respectively), and we observed a CI_50_ value of 0.58, which is indicative of moderate synergy (Figure 6B). Thus, we observed that the combination activity depends on the dose ratio of the drugs and that unique drug-drug activities are observed when delivering drug combinations with polymeric nanocarriers.

Combination effects of TFV with either free or encapsulated SQV were tested at 1∶1 ratios of their IC_50_ values, which corresponded to a 1∶5 TFV∶SQV and 1∶3 TFV∶NP-SQV molar ratio (Figure 6C). We found that free TFV combined with free SQV showed only an additive effect (CI_50_ = 1.04). However, a synergistic effect (CI_50_ = 0.07) was observed from combining of free TFV and nanoparticle-SQV. Collectively, drug synergy data demonstrate that combining free TFV with either NP-EFV or NP-SQV results in pronounced synergistic effects.

## Discussion

Combination drug approaches are an emerging paradigm for the prevention of HIV-1 infection. However, chemical incompatibilities prevent the combination of many existing drugs in a manner that could enhance potency by realizing unique combination drug activities. We hypothesized that polymeric nanocarriers could facilitate the discovery of unexplored drug-drug activities by enabling the combination of chemically incompatible agents. We demonstrate that ARVs can be encapsulated within polymeric nanoparticles to provide synergistic prophylaxis in combination with TFV. We also report on novel combinations of NP-ARVs with TFV that show synergistic anti-HIV activity *in vitro*. Our findings reveal a relevant strategy for delivering multiple ARVs in combination to enhance drug potency, lower cytotoxicity and reduce the likelihood for developing drug resistance. We expect that the versatility of nanoparticle delivery platforms will result in broad applications for HIV chemoprophylaxis and treatment. ARV-encapsulating nanoparticles may overcome barriers in the delivery of agents with diverse physicochemical properties, particularly via administration routes with limited dosage forms for combination drug delivery such as topically to the genital and rectal mucosa.

Our NP-ARVs demonstrate enhanced antiviral activity against HIV-1 BaL compared to unformulated ARVs. Using an *in vitro* TZM-bl indicator cell model previously used to evaluate drug candidates for topical microbicides [37], [38], we observed higher inhibitory activity of NP-EFV than free EFV with a 50-fold reduction in IC_50_. Similarly, NP-SQV showed greater anti-HIV activity when compared to the free drug. NP-ARVs formulated with PLGA have previously been demonstrated to facilitate drug uptake resulting in higher intracellular drug concentrations and greater inhibitory activity [49], [55]. Destache et al. showed that monocyte-derived macrophage (MDM) cells incubated with ARV loaded PLGA nanoparticles exhibited higher intracellular drug concentrations than those incubated with free ARVs. Although we did not measure intracellular uptake of particles in this study, the 50-fold reduction in IC_50_ values between NP-ARVs and free drugs suggests that the particulate nature of our delivery platform plays a major role in improving bioactivity. As Destache et al. observed, it is likely that the PLGA nanoparticles facilitate increased drug uptake and intracellular retention of our ARV drug candidates.

Based on the use of TFV in both oral and topical prophylactic prevention trials, we were motivated to explore the possible enhanced activity when combining free TFV with our NP-ARVs. These dose reduction results indicate that less of each drug was required to inhibit HIV-1 when compared with single-drug use. NP-ARVs alone and in combination mediated even greater dose reduction compared to the free drug equivalents. Therefore, NP-ARVs may be considered superior to free ARVs, since NP-ARVs combined with free TFV provided enhancement in dose reduction up to 600-fold. While dose reduction results can be used to predict the optimal therapeutic doses, they do not provide quantitative information to indicate the degree of synergism [32]. In other words, IC_50_ values obtained from dose-response studies by themselves did not reflect whether the combination effect was additive, synergistic, or antagonistic. This is particularly evident when the IC_50_ values of combined drugs are only slightly different from the single-drug treatments. As found in this study, the IC_50_ values of free TFV combined with either free SQV or NP-SQV were of the same order of potency with an observed 2 to 7-fold dose reduction.

Using the median-effect analysis, we were able to quantify synergistic effects of combined drugs. We found that the combination of free TFV with free EFV at a 1∶1 ratio of IC_50_ values demonstrated a synergistic effect (Figure 6A). Similar synergistic effects were observed when the combination of free TFV and free EFV was tested in MT-2 cells [52]. However, when we used a 1∶1 ratio of IC_50_ values to evaluate the combination effect of NP-EFV with free TFV, we observed only an additive effect (Figure 6B). It may not be expected that free drugs used in combination would result in the same effect as the identical drug combinations delivered using NP-ARVs. Unlike many small molecule drugs whose cellular transport is dominated mostly by passive diffusion, the delivered drug dose from nanoparticles depends on the drug loading and release kinetics as well as the properties of the particles (number, size, shape, chemistry) and cells (density, internalization rate). At the equipotency ratio of NP-EFV and free TFV, the molar drug dose of TFV is in 600-fold excess to EFV delivered via nanoparticles. The molar excess of TFV may actually be higher given that only 10% of the loaded EFV was released from the nanoparticles within the first hour. The effective EFV dose delivered from nanoparticles also depends on the average number of NP-EFV per cell, which is at most ∼6 at the IC_50_ value for the combination dose. Therefore, at the equipotency ratio for NP-EFV and free TFV, these differences may bias the activity of the free drug and explain the observed IC_50_ value and combination effect for NP-EFV and free TFV. When instead we tested the combination effect of NP-EFV and free TFV at the same molar ratio used for the free drug combinations, we observed synergistic activity (Figure 6A). Our result supports observations that, while combination studies are recommended to be performed at the equipotency ratio, other combination ratios may be optimal to achieve maximum synergy. Experiments that evaluate combination effects at all possible combinations using different concentrations of NP-ARVs and free drug may be necessary to inform the use of nanocarriers for combination drug delivery [56].

The combination of NP-SQV and free TFV demonstrated superior action to that of free SQV/free TFV combination (Figure 6C). SQV is the first HIV-protease inhibitor marketed for the treatment of HIV and is currently recommended in combination therapy regimen with nucleoside inhibitors of HIV reverse transcriptase [9], [14]. Nevertheless, its poor absorption is a prominent pharmacologic characteristic of SQV [19], [57], [58], [59]. We demonstrated a greater synergistic effect by combining NP-SQV with free TFV (CI_50_ = 0.07) than that of combining free SQV with free TFV (CI_50_ = 1.04). Previous studies have shown that administration of polymeric nanoparticles significantly increases the intracellular concentration and permeability of SQV [60], [61]. We expect that the improved bioactivity of our NP-SQV is due to similarly increased bioavailability provided by the nanoparticle formulation.

The *in vitro* drug release profile of our NP-ARVs showed an initial burst release within 1 h followed by sustained release of drug over 24 h (Figure 2D). We expected that this biphasic release profile would provide a sufficient amount of rapid release of ARVs to prevent HIV-1 infection soon after application and then sustain the release of drugs for prolonged protection. The *in vitro* release studies from EFV nanoparticles may have been limited by poor aqueous solubility of EFV and small release buffer volumes needed to maintain a concentration detectable by HPLC, resulting in sink conditions not being met. Future release experiments using a cosolvent such as polysorbate 80 or PEG 400 may prove useful in establishing sink conditions [62], [63]. *In vitro* release profiles can be affected by the environmental conditions. As reported by Ham et al., the percent drug release from nanoparticles at pH 7.4 was lower compared to the release profiles obtained at pH 4.6 [37]. While we have not yet investigated *in vitro* release profiles at different pH conditions, further *in vitro* release studies are planned to determine the impact of pH on drug release and how the release patterns can help in determining the synergistic combination ratios.

Currently, nanoparticle encapsulation as a drug delivery system is being investigated in numerous therapeutic fields [17]; however, it is still emerging within the microbicide field [16]. In this study, we provide evidence for the efficacy of a PLGA nanocarrier system to achieve HIV-1 inhibition. Our results suggest that PLGA nanoparticles are a safe delivery platform for encapsulating and delivering ARVs. Furthermore, we show that our NP-ARVs act synergistically with TFV in preventing the infection of HIV-1 *in vitro*. Different models describe the classification of synergy, antagonism, and additive activity arising from the combination of multiple drugs [56]. We applied the approach of Chou and Talalay based on the median-effect principle for mutually exclusive drug combinations. This model is often chosen even though it assumes that the combinations involve multiple drugs competing for the same binding site. In general, the model provides a conservative estimate of inhibition and deviates from the alternative model for nonexclusive or independent drug action only at high drug concentrations [64]. Therefore, we expect that the synergy classification prescribed by our model for NP-ARV combinations to be supported by other methods used for combination studies, and deserves further study. To our knowledge, this work reports the first quantitative measure of synergy by combining ARV-nanoparticles and TFV. Our results also highlight new opportunities to design and quantify combination studies mediated by nanocarrier delivery systems.
